# Long non-coding RNA signature in colorectal cancer: research progression and clinical application

**DOI:** 10.1186/s12935-023-02867-0

**Published:** 2023-02-16

**Authors:** Yudi Wu, Xiangshang Xu

**Affiliations:** 1 GI Cancer Research Institute, Tongji Hospital, Huazhong University of Science and Technology, Wuhan, People’s Republic of China; 2 Department of Gastrointestinal Surgery, Tongji Hospital, Tongji Medical College, Huazhong University of Science and Technology, 1095 Jiefang Avenue, Wuhan, 430030 People’s Republic of China

**Keywords:** colorectal cancer, Long non-coding RNA, Diagnosis, Biomarker

## Abstract

Colorectal cancer is one of the top-ranked human malignancies. The development and progression of colorectal cancer are associated with aberrant expression of multiple coding and non-coding genes. Long non-coding RNAs (lncRNAs) have an important role in regulating gene stability as well as gene expression. Numerous current studies have shown that lncRNAs are promising biomarkers and therapeutic targets for colorectal cancer. In this review, we have searched the available literature to list lncRNAs involved in the pathogenesis and regulation of colorectal cancer. We focus on the role of lncRNAs in cancer promotion or suppression, their value in tumor diagnosis, and their role in treatment response and prognosis prediction. In addition, we will discuss the signaling pathways that these lncRNAs are mainly associated with in colorectal cancer. We also summarize the role of lncRNAs in colorectal precancerous lesions and colorectal cancer consensus molecular subgroups. We hope this review article will bring you the latest research progress and outlook on lncRNAs in colorectal cancer.

## Introduction

Long non-coding RNAs (lncRNAs) are widely found in the human body and are transcripts that regulate many cellular functions [1]. They are generally more than 200 nucleotides in length, but do not have protein-coding capabilities [2]. LncRNAs are abnormally expressed in many kinds of tumors, indicating that they may play a role in regulating tumor pathogenesis [3]. In addition, numerous studies have found that lncRNAs are potential biomarkers in carcinogenesis and are also expected to become markers for tumor treatment and prognosis judgment [4–6].

Colorectal cancer (CRC) is a very common malignancy with the highest incidence and mortality in the world [7]. Many recent studies have shown that the abnormal expression of lncRNA is significantly associated with the risk and prognosis of CRC [8, 9]. The expression levels of many lncRNAs in CRC tumor tissues are correlated with typical tumor characteristics such as depth of invasion, distant metastasis and lymph node metastasis, and are correlated with the overall clinical stage [5, 10–12].

LncRNAs are involved in regulating tumor occurrence and development, so the changes in their expression level also indicate the different states of the disease [13, 14]. Based on this, lncRNAs may also be used as a tumor’s diagnostic and prognostic marker and a possible treatment target. In this review, we will summarize the existing research on the abnormal expression of lncRNAs in CRC, its relationship with prognosis and treatment response, and related signaling pathways, show the progress of lncRNAs in CRC related research and propose relevant prospects.

### Search method

The PubMed database and Web of Science were searched for literature with the keywords *(“colorectal cancer” or “colorectal malignancy”) AND (“long non-coding RNA” or “lncRNA”)* up to 21 Sep. 2022, with a focus on the past 10 years. Articles were those in English relating to colorectal cancer.

### Oncogenic lncRNAs in colorectal cancer

As shown in Table 1, we summarize the well-studied oncogenic lncRNAs, which act on different targets and play different roles in promoting tumor progression. Many studies have shown that their expression is significantly increased in tumor tissues compared with normal tissues, and patients with high expression of these lncRNAs often have poor prognoses. Among them, the most representative lncRNAs are colorectal cancer associated transcript (CCAT) [4, 10, 15–19], H19 [11, 20, 21], lung adenocarcinoma transcript 1 (MALAT-1) [5, 22] and HOTAIR [23, 24].

**Table 1:** Oncogenic lncRNAs in colorectal cancer (OS: overall survival, DFS: disease free survival)

LncRNA	Chromosomal location	Signaling pathway	Number of cases	Patient outcome	Function	References
AP002754.2	11q12.2	E2F1/FADS2	67 CRC vs 14 adjacent	–	promote CRC progression	[25]
CASC11	8q24	Wnt/β-catenin	36 paired CRC vs adjacent	–	promote CRC growth and metastasis	[26]
CCAL	8q24.21	Wnt/β-catenin	131 stage II vs 74 stage III CRC	Shorter OS	promote CRC progression	[4]
CCAT1-L	8q24.21	CCAT1*/*MYC	–	–	promote CRC progression	[15]
CCAT1 and CCAT2	8q.24.21	–	280 CRC vs 20 adjacent	Shorter OS and DFS	promote CRC proliferation	[17]
CCAT2	8q24.21	CCAT2/miRNA-145*/*MYC	–	–	promote CRC progression	[16]
CCAT2	8q24.21	CCAT2/MYC; Wnt/β-catenin	215 CRC vs 94 normal	Shorter OS	promote CRC metastatic and chromosomal instability	[10]
CCAT2	8q.24.21	CCAT2/MYC and BOP1/AURKB	900 CRC different stages	Shorter OS and DFS	induce chromosomal instability	[19]
CRNDE	16q12.2	Wnt/β-catenin	64 paired CRC vs normal	Shorter OS	promote CRC proliferation and chemoresistance	[27]
CYTOR	2p11.2	NF-κB signaling	138 paired CRC vs normal	Shorter OS and DFS	promote CRC progression	[28]
DLEU1	13q14.2–14.3	DLEU1/SMARCA1/KPNA3	100 paired CRC vs normal	Shorter OS and DFS	contribute to CRC progression	[29]
ENO1-IT1	1p36.23	KAT7/ENO1/IT1	173 CRC and 116 paired CRC vs normal	–	promote glycolysis and oncogenesis	[30]
EVADR	6q13	EMT	59 CRC with vs 34 without metastasis	Shorter OS	promote CRC metastasis	[31]
FEZF1-AS1	7q31.32	FEZF1-AS1/PKM2	108 paired CRC vs normal	Shorter OS	promote CRC proliferation, metastasis	[32]
FLANC	22q13.31	STAT3/VEGFA	56 paired CRC vs normal	Shorter OS	promote angiogenesis	[8]
GLCC1	8q24.21	GLCC1/c-Myc/LDHA	95 paired CRC vs adjacent	Shorter OS	promote carcinogenesis	[9]
GSEC	11q24.2	–	22 paired CRC vs adjacent	–	modulate CRC cell migration	[33]
H19	11p15.5	Wnt/β-catenin pathway	20 paired CRC vs adjacent	–	promote the stemness and chemoresistance	[20]
H19	11p15.5	Rb/E2F and CDK8/β-Catenin	700 CRC different stages	Shorter OS and DFS	promote CRC stemness and chemoresistance	[21]
H19	11p15.5	H19/hnRNPA2B1/EMT	11 CRC with vs 49 without metastasis	Shorter OS	promote CRC metastasis and EMT	[11]
HOTAIR	12q13.13	JAK2/STAT3	95 CRC different stages	Shorter OS	impact CRC metastasis, tumorigenesis	[23]
HOTAIR	12q13.13	microRNA-93/ATG12	42 paired CRC vs adjacent	Shorter OS	CRC tumorigenesis and radioresistance	[24]
KRT7-AS	12q13.13	KRT7-AS/KRT7-κB	49 CRC different stages	–	promote CRC metastasis	[34]
LINC00152	2p11.2	YAP1/LINC00152/FSCN1	83 paired CRC vs adjacent	Shorter OS	promote CRC progression	[35]
Linc00659	20q13.33	PI3K/AKT	89 paired CRC vs adjacent	Shorter OS	promote CRC growth	[36]
LINRIS	3q27.2	LINRIS/IGF2BP2/MYC	118 paired CRC vs adjacent	Shorter OS	promote CRC aerobic glycolysis	[37]
Lnc-FAM84B-4	5q35.1	MAPK signaling	72 paired CRC vs adjacent	–	promote CRC proliferation, metastasis	[38]
LncSLCC1	1q41	SNP/lncSLCC1/AHR/HK2	720 CRC different stages	Shorter OS	promote CRC progression	[39]
LOC90024	4q24	SRSP/ SRSF3/Sp4	101 CRC vs 79 normal	Shorter OS	promote CRC tumorigenesis	[40]
LUCAT1	5q14.3	LUCAT1/PTBP1	168 paired CRC vs adjacent	Shorter OS	modulate CRC viability and chemotherapy response	[41]
MALAT1	11q13.1	MALAT1/miR-106b-5p/SLAIN2	168 paired CRC vs adjacent; 95 CRC different stages	Shorter OS and DFS	promote CRC invasion and metastasis	[22]
MALAT1	11q13.1	MAP4K4/c-Jun/MDM2	1078 CRC vs 1175 normal	Shorter OS	promote CRC growth and metastasis	[5]
MIR100HG	11q24.1	Wnt/β-catenin	359 CRC different stages	–	cetuximab resistance	[42]
MIR17HG	13q31.3	NF-κB/RELA	6 CRC vs 6 adenoma; 809 CRC different stages	Shorter OS	promote CRC progression	[43]
MNX1-AS1	7q36.3	MYC/MNX1-AS1/YB1	21 paired CRC vs adjacent 150 CRC vs 111 normal	Shorter OS	promote CRC progression	[44]
NEAT1	11q13.1	miR-34a/SIRT1; Wnt/β-catenin	30 paired CRC vs adjacent	–	promote CRC progression	[45]
NEAT1	11q13.1	NEAT1/DDX5/Wnt/β-catenin	718 CRC vs 112 normal	Shorter OS and DFS	promote CRC progression	[46]
N-BLR	Xp13	N-BLR/ZEB1	284 paired CRC vs normal	Shorter OS	promote invasion and migration	[47]
PiHL	8q24.21	GRWD1/RPL11/MDM2	83 paired CRC vs adjacent	Shorter OS	promote CRC progression	[48]
PINCR	Xp11.3	p53 network	–	–	promote CRC progression	[49]
PURPL	5p14.1	PURPL/MYBBP1A/p53	79 paired CRC vs adjacent	–	promote CRC tumorigenesis	[50]
PVT1	8q24.21	TGFβ/SMAD; Wnt/β-catenin	36 paired CRC vs adjacent; 698 CRC different stages	Shorter OS	promote CRC progression	[12]
PVT1-214	2q21.3	Lin28/let-7	92 paired CRC vs adjacent	Shorter OS and DFS	promote CRC proliferation, invasion	[51]
RAMS11	6p12.1	RAMS11/ CBX4/ TOP2α	232 CRC different stages	Shorter OS	promote CRC metastasis	[52]
RP11	19q13.42	EMT	442 CRC different stages	Shorter DFS	trigger the CRC dissemination	[53]
SNHG5	6q14.3	STAT3 signaling	324 CRC vs 72 adenomas, 314 normal tissues	–	promote CRC survival	[54]
SNHG6	8q13.1	SNHG6/EZH2	–	–	promote CRC growth and metastasis	[55]
SNHG7	9q34.3	PI3K/Akt/mTOR pathway	70 paired CRC vs adjacent	Shorter OS and DFS	promote CRC proliferation, metastasis	[56]
UICLM	2q37.3	UICLM/miR-215/ZEB2	7 CRC with vs 8 without liver metastasis; 122 CRC tissues different stages	Shorter OS	promote CRC liver metastasis	[57]
UPAT	19p13.3	UHRF1/UPAT	–	–	promote CRC tumorigenesis	[58]
XIST	Xq13.2	METTL14/YTHDF2	37 paired CRC vs adjacent	Shorter OS	promote CRC proliferation, metastasis	[59]

CCATs are a series of the earliest identified long noncoding RNAs in CRC, which are located at chromosome 8q24. The findings of CCAT1 and CCAT2 demonstrated the critical role of lncRNAs in CRC and emphasized the need for comprehensive lncRNA analysis in CRC [10, 60]. Ma and colleagues demonstrated that CCAL could promote the development of CRC by targeting and activating AP-2α, and the specific mechanism is that CCAL could induce multidrug resistance (MDR) by activating the Wnt/β-catenin signal pathway by inhibiting AP-2α and further promoting MDR1/P-gp expression [4]. Xiang et al. revealed that CCAT1-L is site-specific transcribed upstream of MYC in human colorectal cancer and that CCAT1-L plays a role in the regulation of MYC transcription and promotes long chromatin looping. [15]. Since CCAT-1 was upregulated in both pre-malignant and malignant conditions in CRC [60], it might become a highly specific biomarker for CRC [61]. The study of Yu et al. explained the new mechanism of lncRNA-miRNA in CRC, and they proved that CCAT2 could selectively block miR-145 maturation by suppressing pre-miR-145 export to cytoplasm and block the cleavage of pre-miR-145 by Dicer [16]. These results reveal that CCATs could promote CRC progression through different mechanisms.

H19 is oppositely expressed to IGF2, located at chromosome 11p15.5 [62]. In most human tissues, IGF2 imprinting depends on a differentially methylated region (DMR) upstream of H19 [63]. Aberrant DNA methylation can alter the expression of imprinted genes, which may be one of the causes of CRC [64]. In 2004, Fellig et al. found that lncRNA H19 was overexpressed in liver metastases of CRC [65]. Studies have found that H19 can promote the stemness of cancer stem cells (CSCs) and chemoresistance of CRC cells in CRC, and the mechanism is that CAFs could promote CRC stemness and chemoresistance by transferring H19 in exosome, H19 could activate the β-catenin signaling trough acting as a competing endogenous RNA sponge for miR-141, while miR-141 has the function to inhibit CRC stemness [20]. In addition, Ohtsuka et al. showed that H19 could affect RB1 phosphorylation by regulating CDK4 and CCND1 expression, and interact with CDK8 gene transcription regulator macroH2A to regulate CDK8 expression and influence β-catenin activity to promote the progression of CRC [21]. The work of Zhang et al. [11] reported that lncRNA H19 was significantly overexpressed in CRC primary tumors and metastatic tissues, which significantly correlates with a poor prognosis of CRC. Mechanistically, ectopic H19 expression promotes CRC cells’ metastasis and induces epithelial to mesenchymal transition (EMT) by binding nuclear hnRNPA2B1which could activate Raf-ERK signaling.

MALAT-1 is a lncRNA expressed from chromosome 11q13 and was first found overexpressed in non-small cell lung cancer [66]. In 2011, Xu et al. proved the importance of MALAT-1 in CRC metastasis; they analyzed the MALAT-1 gene expression in five fragments and found that one fragment (6918 nt-8441 nt) located at the 3’ end of MALAT-1 plays an important role in CRC cell proliferation, migration and invasion [67]. Additionally, Zhu et al. showed that MALAT-1 was expressed highly in CRC tissues, and high MALAT-1 expression levels may be a poor prognostic marker in stage II and III CRC patients [68]. Recent studies have demonstrated that MALAT-1 promoted CRC growth, invasion and metastasis [5, 22]. Ji and colleagues demonstrated that knockdown of MALAT-1 in CRC cells was able to inhibit the transfer of β-catenin between the cytoplasm and nucleus, which resulted in reduced expression of c-Myc and MMP-7 and a decrease in invasive and metastatic ability [69]. These results suggest that MALAT1 is involved in the regulation of CRC invasion and metastasis by regulating the WNT/β-catenin signaling pathway.

HOTAIR is transcribed at the HOXC locus on chromosome 12q13.13, opposites to the *HOXC* gene. Kogo et al. found that the expression level of HOTAIR in CRC tissues was higher than that in normal tissues, and a close association was found between HOTAIR expression and PRC2 complex (SUZ12, EZH2 and H3K27me3) using cDNA array analysis [70]. Recent studies proved that HOTAIR promoted CRC progression by mediating lung and liver metastasis and drug resistance [23, 24].

### Tumor suppressor lncRNAs in colorectal cancer

As shown in Table 2, most tumor suppressor lncRNAs are low expressed in tumor tissues, except MIR4435-2Hg. High expression of these lncRNAs often indicates a better prognosis. Studies focusing on tumor suppressor lncRNAs are less common than those focusing on tumor-promoting lncRNAs, and the most common mechanisms of tumor suppressor lncRNAs include inhibiting CRC cell growth [71–74], invasion [75] and metastasis [72, 76, 77].

**Table 2:** Tumor suppressor lncRNAs in colorectal cancer (OS: overall survival, DFS: disease free survival)

LncRNA	Chromosomal location	Signaling pathway	Number of cases	Correlation with outcome	Function	References
GAS5	1q25.1	Hippo/YAP	208 paired CRC vs normal; 183 CRC different stages	Better OS	Inhibit CRC progression	[78]
LINC01133	1q23.2	EMT	219 paired CRC vs adjacent; 776 CRC different stages	Better OS, DFS	Inhibit CRC EMT and metastasis	[76]
LINC02023	3q26.1	PTEN pathway	60 paired CRC vs adjacent; 776 CRC different stages	–	Inhibit CRC cell proliferation	[71]
LINC00675	17p13.1-p12	BRI3BP/FLAG/Myc	83 CRC vs 79 normal	–	Inhibit CRC tumorigenesis	[64]
lncRNA-APC1	3q26	APC/PPARα	110 CRC vs 30 normal	Better OS	Inhibit CRC pathogenesis	[79]
MEG3	14q32.3	VDR/MEG3/Clusterin	27 paired CRC vs normal	Better OS	Represses CRC proliferation	[72]
MIR4435-2HG	2q13	PMN/MDSCs	10 paired CRC vs normal	Better OS	Suppress CRC progression	[80]
OCC-1	12q23.3	OCC-1/HuR	919 CRC different stages	–	Suppress CRC progression	[74]
OLA1P2	17p11.2	FOXD3/OLA1P2/STAT3	196 CRC different stages	Better OS	Inhibit CRC progression	[81]
SATB2-AS1	2q33.1	SATB2/Snail	40 CRC vs 30 normal	Better OS	Suppress CRC aggressiveness	[75, 77]

As a well-characterized tumor suppressor, PTEN is crucial in the initiation and progression of various cancers. Focus on the PTEN-dependent signal pathway, Wang and colleague showed that Linc02023 suppressed tumorigenesis of CRC by regulating PTEN stability [71]. In one study, LINC01559 was found to be downregulated in CRC compared to normal tissues and lower expression of LINC01559 in CRC patients indicated a poor prognosis; this study revealed the mechanism as negative regulation of the LINC01559/miR-106b-5p/PTEN axis in CRC progression and disclosed a new mechanism of METTL3-mediated m6A modification on LINC01559 [82].

EMT is one of the important mechanisms of CRC metastasis. By acting on different targets, tumor suppressor lncRNAs could inhibit CRC metastasis by prohibiting EMT process. Therefore, lncRNAs associated with EMT are gaining attention. Kong and colleagues [76] reported that LINC01133 inhibited EMT and metastasis in CRC by acting on SRSF6 and SRSF6 was proven to promote EMT and metastasis in CRC cells. Additionally, Wang et al. found that the expression of lncRNA SATB2-AS1 in CRC tissues was significantly lower than in normal tissues, and its expression was related to tumor staging and prognosis [75]. Further studies showed that SATB2-AS1 overexpression inhibited *CRC* cell proliferation, invasion and migration in vivo and in vitro*.* Another study showed that SATB2-AS1 could inhibit metastasis of CRC cells and regulates Th1-type chemokine expression and immune cell density in CRC tissues; SATB2-AS1 could directly bind to WDR5 and GADD45A, and mediate the deposition of H3K4me3 in the SATB2 promoter region and DNA demethylation, thereby activating SATB2 cis-transcription [77].

In 2022, Yu et al. reported by GEO and TCGA database analysis that the expression of lncRNA MIR4435-2HG in CRC tumor tissues was significantly higher than that in normal tissues [80]. Further experiments demonstrated that MIR4435-2HG is a tumor suppressor gene that regulates the immune microenvironment. MIR4435-2HG deletion resulted in reduced neutrophils and elevated polymorphonuclear myeloid-derived suppressor cells (PMN-MDSCs). In tissue-specific MIR4435-2HG knockout mice, MIR4435-2HG deletion in neutrophils was confirmed to enhance the immunosuppressive capacity of PMN-MDSCs by interfering with their fatty acid metabolism and promoting the progression of colorectal cancer [80].

### LncRNAs as potential diagnostic markers in colorectal cancer

Since lncRNAs are involved in regulating CRC development, the expression of lncRNAs varies in different disease states. According to the abnormal expression of lncRNA in CRC tissues or blood, it may become a new indicator for tumor diagnosis (Table 3).

**Table 3:** Potential clinical diagnosis implication of lncRNAs in colorectal cancer

LncRNA	Number of cases and samples	Detection method	Potential clinical diagnosis implication	References
FLANC	56 Paired CRC vs normal tissues or colitis	FISH	Early diagnosis, distinguish tumor from IBD	[8]
N-BLR	284 Paired CRC vs normal tissues or colitis	FISH		[47]
MIR17HG	6 CRC vs 6 adenoma tissues; 809 CRC different stages	qPCR	Early diagnosis, distinguish adenoma and adenocarcinoma	[43]
SNHG5	324 CRC vs 72 adenomas, 314 normal	qPCR		[54]
NEAT1	718 CRC vs 112 normal tissues	qPCR	Early diagnosis	[46]
PVT1	36 Paired CRC vs adjacent tissues; 698 CRC different stages	methylation array		[12]
PVT1-214	92 Paired CRC vs adjacent tissues	qPCR		[51]
RP11	442 CRC different stages	qPCR		[53]
H19	700 CRC different stages	qPCR	Predict different CRC stages	[21]
MALAT1	168 Paired CRC vs adjacent; 95 CRC different stages	qPCR		[5, 22]
MIR100HG	359 CRC different stages	qPCR		[42]
UICLM	7 CRC with vs 8 without liver metastasis; 122 CRC different stages	qPCR	Predict CRC metastasis	[57]
KRT7-AS	49 CRC different stages	qPCR		[34]
CCAT2	280 CRC vs 20 adjacent tissues	qPCR		[16, 17]
EVADR	59 CRC with vs 34 without metastasis	qPCR		[31]
GLCC1	95 Paired CRC vs adjacent tissues	qPCR	Serve as indicators of prognosis	[9]

Several articles have evaluated the action of lncRNA in CRC diagnosis. For example, Chen’s study reported that: the later the clinical stage of CRC patients, the higher the expression level of lncRNA UICLM in the tumor tissues. At the same time, the expression of UICLM in tumor tissues of patients with liver metastases was significantly higher than that without liver metastases [57]. Therefore, UICLM expression in tumor tissues can be used as a potential predictor of tumor stage and liver metastasis. Xu et al. found that MIR17HG expression in colorectal adenocarcinoma was higher than that in normal tissues and adenomas, which indicated that MIR17HG might be one of the indicators for the early diagnosis of colorectal cancer to distinguish adenoma from adenocarcinoma [43]. One study found that: Compared with healthy people, the methylation level of Plasmacytoma Variant Translocation 1 (PVT1) at CpG site (CG23898497) was higher in CRC patients, and the AUC values of the two cohorts were 0.99 and 0.81, respectively. Therefore, it is considered as a diagnostic marker of CRC [12]. As reported by Wang et al., Lnc34a expression was significantly higher in specimens with advanced CRC than that from earlier tumors, suggesting that Lnc34a expression may be a potential biomarker for advanced stages [83]. To assess the relationship between FLANC expression levels and CRC, Pichler et al. performed in situ hybridization (ISH) using tissue microarrays and found that: overall, FLANC expression was significantly higher in adenocarcinoma and metastatic lesions than in normal, benign polyp and inflammatory colon tissues; however, there were no significant differences between inflammatory colon tissues and polyp lesions and normal tissues; this suggests that upregulation of FLANC may occur in malignant epithelial cells and therefore FLANC may be a sensitive indicator for early diagnosis of CRC and may specifically distinguish tumor from benign disease [8]. N-BLR was found to have similar detection efficiency as FLANC [47]. Damas et al. found that SNHG5 was significantly upregulated from normal to adenoma and to stage I cancer tissues, suggesting that different SNHG5 expression levels could become an early predictive marker for CRC progression [54]. Zhuang and colleagues revealed that the expression of MALAT1 was directly associated with CRC pathology stage and tumor recurrence, indicating that the high expression of MALAT1 may be used as a marker of advanced CRC and recurrence [22]. A study showed that the expression levels of MIR100HG differed significantly in stage I, II and III/IV CRC tissues, suggesting that MIR100HG may be one of the markers for predicting CRC tumor stage; MIR100HG’s function is mediated by let-7a-2–3p, miR-125b-5p, and miR-125b-1–3p expression, all encoded by intron-3 [42]. RP11 was also detected in different stages of CRC and normal tissues with significantly different results, and it has good specificity in both CRC cell line and clinical tissues, meaning that RP11 might be one of the new markers for early diagnosis [53]. Tang et al. reported that combining GLCC1 expression and TNM staging resulted in a more accurate prognosis of CRC (Mixed_AUC_ = 0.76, TNM_AUC_ = 0.70) [9]. The expression of KRT7-AS is significantly different in different stage N CRC patients, indicating that KRT7-AS may be used to predict the lymph node metastasis of CRC [34]. Wu et al. showed that NEAT1 in blood had high sensitivity and specificity in distinguishing colorectal cancer patients from normal control patients, both of which were 83.3%, indicating that NEAT1 could provide an important basis for early screening of CRC [84]. One study found that PVT1-214 showed significant predictive significance in distinguishing CRC from normal tissue, and the area under the ROC curve was 0.803 [51]. Other studies revealed the potential of H19 to predict different CRC stages [21], CCAT2 [10, 17] and EVADR [31] to predict CRC metastasis.

### LncRNAs’ functions in prognosis evaluation and therapeutic guidance

The prognosis of CRC has long been dependent on classic TNM staging. However, we found that the current stage does not reflect the actual survival status of CRC patients well, and there is a complex interaction between the pathological stage and biomarkers to guide the prognosis, risk stratification and predict the treatment effect [85]. Therefore, to make more accurate prognostic predictions, many research teams have begun to focus on finding new markers to combine with or replace CRC TNM staging. Many articles have reported that the abnormal expression of some lncRNAs is closely related to the poor prognosis of CRC patients (Table 4).

**Table 4:** Prognosis evaluation and therapeutic guidance value of lncRNAs in colorectal cancer

LncRNA	Prognosis evaluation	Therapeutic guidance	References
CCAL	High CCAL expression → shorter OS	Low CCAL expression → chemotherapy should be performed cautiously	[4]
FLANC	High FLANC expression → shorter OS and DFS	siRNA therapeutics targeting FLANC leads to decreased proliferation and metastasis in vivo	[8]
SNHG5	–	High SHNG5 expression → drug resistance	[54]
GLCC1	High GLCC1 expression → shorter OS and DFS	–	[9]
MALAT1	No-relapse group → less predictive significance; relapse group → shorter OS and DFS	–	[5]
MIR17HG	High CCAL expression → shorter OS	–	[43]
NEAT1	High NEAT1 expression → shorter OS and DFS; had synergistic effects with DDX5	–	[46]
PVT1	High PVT1 expression → shorter OS and DFS	–	[12]
RAMS11	High RAMS11 expression → shorter OS and DFS	–	[52]

Treatment strategies for patients with stage II and III CRC after radical surgery are very controversial because only part of patients is effective with chemotherapy. Therefore, there is an urgent need for more stringent biomarkers to help screen these patient groups [86]. Ma et al. found that: high expression of CCAL was associated with poor prognosis in patients with stage II and III CRC; patients with high CCAL expression could obtain good curative effects from chemotherapy; however, patients with low CCAL expression had poor chemotherapy response; these results indicate that CCAL expression level can be used as a marker to evaluate the prognosis of CRC patients and whether it is effective for chemotherapy [4]. Xu and his colleagues recruited a total of 752 patients and showed that: 5 year survival in patients with high MIR17HG expression was less than 10%, while 5 year survival in patients with low MIR17HG expression was more than 60%; thus, MIR17HG expression had an important role in predicting patient prognosis [43]. Using results from four different cohorts, Zhang’s study demonstrated that high expression of NEAT1 in CRC predicted a poor prognosis and synergistic with DDX5; in all four cohorts, the 5 year OS for patients was negative for both NEAT1 and DDX5 was > 90% [46]. Pichler et al. found a correlation of lncRNA FLANC expression with CRC prognosis in two independent cohorts and confirmed this in the TCGA dataset and animal experiments [8]. Zhuang et al. demonstrated in a different cohort study that high MALAT1 expression was an important predictor of prognosis in CRC patients with recurrence (P = 0.0167) and had no predictive value for prognosis in patients without recurrence [22]. Tang et al. reported that 5 year OS was 75.6% and 48.9% in the GLCC1^low^ expression group and GLCC1^high^ expression group, and that GLCC1^high^ was related to the poor prognosis of CRC patients [9]. Another study has revealed a similar prognosis evaluation function of lncRNA RAMS11 [52].

### Connection between lncRNAs and colorectal cancer-related signaling pathways

As mentioned above, many lncRNAs play a regulatory role in colorectal cancer-related signaling pathways. In Table 5, we summarized the most frequently investigated signaling pathways associated with lncRNAs. Many reports have shown that abnormally activated Wnt/β-catenin signaling plays an important role in the development of CRC [87, 88]. Ma et al. reported that lncRNA CCAL could activate the Wnt/β-catenin signal through inhibition of AP-2α and regulates MDR1/p glycoprotein expression and thus CRC development by activating the Wnt signaling pathway, MDR1/p glycoprotein was thought to be strongly related to multidrug resistance [4]. Moreover, other studies by Zhang [26], Han [27], Lu [42] and Ren [20] also discovered the regulatory functions in Wnt/β-catenin signaling of several lncRNAs, including CASC11, CRNDE, MIR100HG and H19.

**Table 5:** The connection between lncRNAs and colorectal cancer-related signaling pathways

Signaling pathways	LncRNA	Mechanism of action	Reference
JAK/STAT	FLANC	Induce a higher level of the phosphorylated STAT3	[8]
	SNHG5	Block the action of STAU1	[54]
NF-κB	MIR17HG	Transcriptionally activated by NF-κB/RELA	[43]
	CYTOR	Interacting with NCL and Sam68	[28]
PI3K/AKT	SNHG7	Act as a target of miR-34a to increase GALNT7 level	[56]
	Linc00659	Promote the PI3K-AKT-GSK3B	[36]
TGF-β	PVT1	Associated with genes within the TGFβ/SMAD pathways	[12]
VEGF	FLANC	Moderate VEGFA protein expression	[8]
Wnt/β-catenin	CCAL	Suppress AP-2α	[4]
	NEAT1	Interact with DDX5	[45, 46]
	PVT1	Associated with genes within the Wnt/β-catenin pathways	[12]
	CASC11	Interact with hnRNP-K	[26]
	CRNDE	Moderate the expression levels of miR-181a-5p	[27]
	MIR100HG	Derive miR-100 and miR-125b	[42]
	H19	Act a competing endogenous RNA sponge for miR-141	[21]

LncRNAs are also involved in regulating several other signaling pathways in CRC. For instance, the lncRNA UICLM could induce its biological effects by regulating ZEB2, and ZEB2 depletion inhibits UICLM-promoted tumorigenesis [57]; MIR17HG promotes CRC progression via NF-κB/RELA pathway [43]; SNHG7 can compete against endogenous miR-34a and GALNT7, thereby activating PI3K/Akt/mTOR pathway to promote CRC proliferation and metastasis [56]; FLANC [8] and SNHG5 [54] are involved in STAT3 pathway for similar pathogenic functions.

In many other malignancies, some CRC-related lncRNAs have also been found to regulate PI3K/Akt, Wnt/β-catenin and other signaling pathways [89]. Therefore, more studies are needed to discover the specific roles of these lncRNAs in CRC in relation to these signaling pathways, and these lncRNAs and related signaling pathways can potentially become new targets for colorectal cancer therapy in the future.

### LncRNAs with colorectal precancerous lesions

Studies have shown that most CRC can be prevented by early detection and removal of precancerous lesions [90, 91], suggesting the relationship between precancerous lesions and CRC. In recent years, there has been increasing interest in studying tumorigenesis and new markers (*e.g.,* membrane proteins, miRNAs and lncRNAs).

Various lncRNAs have been confirmed to be closely related to the occurrence and development of colorectal adenoma [92]. LncRNAs expression can be disrupted in CRC through several mechanisms, such as hypermethylation of its promoters’ CpG islands (CpGIs) [93]. Ruiz-Bañobre et al. [94] found that the methylation level of LINC00473 in CRC and polyps tissues was significantly higher than in normal controls, while there was no significant difference between CRC and polyps tissues. Moreover, LINC00473 methylation levels were significantly different in a non-advanced colorectal polyp (N-ACP) compared with advanced colorectal polyps (ACP) and CRC. These findings suggested that the altered expression of LINC00473 might promote the transformation of normal tissues into precancerous lesions. Through clinical testing, Xu et al. [95] found that SNHG11 showed strong diagnostic ability in identifying precancerous lesions and early tumor formation, and they also verified that high expression of SNHG11 promoted cell proliferation and metastasis by targeting the Hippo pathway. Besides, CCAT1 was found to be significantly upregulated at all stages of the adenoma-carcinoma cascade in tumors (fivefold), adenomatous polyps (100-fold), or metastases (more than 100-fold) compared with precancerous mucosa [96]. Siskova et al. [97] found that MALAT1 was related to cancer cell metastasis and migration, and its gene amplification was also an important event in the occurrence and development of colorectal adenoma. One study [98] used Human Transcriptome Array (HTA) 2.0 to analyze LncRNA expression levels in colon biopsy specimens from 20 CRCs, 20 adenomas, and 20 healthy controls, and the results showed that 16 lncRNAs displayed diverse expressions from early carcinogenesis to CRC formation, the lncRNAs included the down-regulated LINC02023, MEG8, AC092834.1 and the up-regulated CCAT1, CASC19. Shaker et al. [99] used real-time PCR to detect LINC00657 expression in serum in CRC, adenomatous polyposis, and ulcerative colitis patients and found that upregulation of LINC00657 was significantly associated with CRC. In addition, serum LINC00657 could distinguish patients with adenomatous polyposis and ulcerative colitis from healthy controls.

### LncRNAs with consensus molecular subgroups

One of the most important features of cancer is genomic instability. In recent years, consensus molecular subgroups (CMS) based on gene expression analysis have received increasing attention in cancer research [100]. There were four different CMSs in CRC: CMS1 is defined by immune activation, which is strongly associated with microsatellite instability (MSI); CMS2 reflects marked canonical pathway activation, which has a close relationship to the mutations of *APC, p53* and *RAS*; CMS3 is defined by metabolic disorders, especially in lipogenesis and glutaminolysis; and CMS4 is defined by activated tissue growth factor-β (TGF-β), stromal invasion and angiogenesis. In the study of CRC CMS, the most talked about are MSI and RAS mutations [100, 101].

Qiu et al. [102] used CRC data from The Cancer Genome Atlas (TCGA) and developed a new deep-learning framework to predict MSI status based on hematoxylin and eosin (H&E) stained images. Their results showed significant differences in mRNA, miRNA and lncRNA between the high microsatellite instability (MSI-H) patient group and the low microsatellite instability or microsatellite stability (MSI-L/MSS) group. Zhu and colleagues [103] used the TCGA database to construct a 15 lncRNAs prognostic model, a novel cuproptosis-related prognosis model and molecular signature associated with prognosis, immune infiltration and immunotherapy. Li et al. [104] found that LINC00963 was overexpressed in several tumor types, including CRC, and high levels of LINC00963 expression were associated with increased copy number, MSI-H and immunotherapy response. One research revealed [105] that differentially expressed lncRNAs (including lncGLI3-4:1, lncCDK5-2:11, lncZG16-1:1 and lncSOD3-3:1) were remarkably correlated with the clinical characteristics of pMMR colon cancer (CC) patients and may be engaged in the tumorigenesis of pMMR CC.

*KRAS* mutations cause approximately 45% of CRC [106]. Saliani et al. [107] identified 12 different expressed lncRNAs (including SSTR5-AS1, RASSF8-AS1, PROSER2-AS1, etc.) that may regulate genes primarily enriched in the Rap1 and RAS signaling pathways and result in a poorer prognosis. Anti-epidermal growth factor receptor (EGFR) monoclonal antibodies (cetuximab and panitumumab) are important for patients with wild-type *RAS* and *BRAF* metastatic CRC, high expression of lncRNAs (MIR100HG, UCA1, CRART16, SLCO4A1-AS1 and TTN-AS1) in CRC enhances the sensitivity of CRC cells to cetuximab and panitumumab [108].

## Discussion

The expression profile of some lncRNAs changes extensively during the pathogenesis of CRC. Much evidence has demonstrated the relationship between lncRNA expression levels and CRC clinicopathological factors. Associations between UICLM expression and TNM stage [57], different levels of PVT1 methylation and TNM stage [12], MALAT1 expression and CRC non-relapse/relapse [22], KRT7-AS expression and lymph node involvement [34] are typical examples. With this, these lncRNAs may better distinguish different clinical stages than traditional classification predicts in a more efficient way.

Chemotherapy is an important tool in the treatment of CRC, but many patients are not sensitive to chemotherapy, which means that this group of patients who are not sensitive to chemotherapy will not have a good therapeutic effect but will suffer from many side effects. Therefore, assessing the response to chemotherapy and finding the sensitive and insensitive groups for targeted treatment is very important. Several studies have shown that lncRNAs play a role in resistance to conventional chemotherapy, so targeting these lncRNAs may yield surprisingly good results [4, 41, 54]. On the other hand, therapeutic targeting of some lncRNAs is considered as a way to improve the efficacy of immunotherapy. LncRNAs characterization will help to select suitable patient groups for therapeutic modalities. Therefore it can provide a protocol design for precise individualized treatment.

Many studies have shown that lncRNA has the potential ability to be a diagnostic or prognostic tool in various tumors. LncRNAs are widely present in tissues, blood and body fluids, and their expression in the blood can always reflect the expression of tissues with a good consistency feature [109, 110]. Detection of lncRNAs is easy to obtain specimens by drawing peripheral blood or examining other body fluids, which is one of the main advantages of lncRNAs as biomarkers for cancer detection [22, 47]. Moreover, the detection method for lncRNAs is mainly performed by PCR, which is a routine method widely used in medical testing and has the characteristics of rapidity, high sensitivity and specificity, and is relatively low cost and easy to carry out. Therefore, the detection of lncRNAs is an ideal tool for tumor diagnosis and prognosis determination.

LncRNAs have the function of regulating genes from upstream and they themselves do not need to be translated into proteins to function. The design of interventions for lncRNAs is relatively simple, and siRNA vector systems that have been widely studied in clinical applications can achieve targeted interventions for lncRNAs [16, 22]. Targeting lncRNAs can regulate tumor signaling pathways or key oncogenes from upstream, which can be used for targeted therapy against tumors. Therefore, intervention in CRC-related lncRNAs or lncRNA-related signaling pathways, would be potential targets for CRC precise therapy.

In conclusion, lncRNAs are considered to be potential markers for early diagnosis of CRC, for determining prognosis, and for understanding response to therapy. It could be used to target treatment options or improve response to conventional therapy. However, before these are realized, a full understanding of the specific functional mechanisms of lncRNAs in CRC is very important and urgent and is a major challenge for implementing lncRNAs in clinical practice.

## Data Availability

The analyzed data sets generated during the study are available from the corresponding author on reasonable request.
